# *“Bad timing for illness relapse!”* Mood symptoms, challenges and strategies for wellbeing in the first year postpartum among infant mothers with bipolar disorder: a mixed-methods study

**DOI:** 10.1186/s40345-025-00374-x

**Published:** 2025-02-24

**Authors:** Teija MS Anke, Dag Vegard Skjelstad

**Affiliations:** Division of Mental Health and Addiction, Vestre Viken Hospital Trust, Drammen, 3004 Norway

**Keywords:** Bipolar disorder, Perinatal disorder, Perinatal mental health services, Postpartum, Infant mother, Maternal severe mental illness, Mood episodes, Bipolar disorder challenges, Wellbeing strategies, Mixed methods

## Abstract

**Background:**

The postpartum period is associated with a high risk of illness episodes in women with bipolar disorder (BD) and is a critical developmental phase for both a new mother and her infant. This mixed-methods study aimed to investigate the occurrence of mood symptoms among infant mothers with BD in the first year postpartum, as well as their perceptions of the first year, their challenges and their strategies for wellbeing.

**Methods:**

Twenty-six women with BD participated. Mood symptoms were assessed at 3 and 12 months postpartum with the Inventory of Depressive Symptomatology and Young Mania Rating Scale. Occurrences of additional postpartum mood deviations were investigated through an interview at 12 months, which also covered the women’s postpartum experiences. Thematic analysis was applied to the qualitative dataset (interviews and field notes).

**Results:**

42% of the women were euthymic or had only mild mood symptoms at 3 and 12 months. 58% had moderate to severe symptoms at either or both time points. A positive (38%) vs. mixed (62%) perception of the first year was strongly associated with euthymia-mild vs. moderate-severe mood deviations, as was the experience of maternal developmental achievement vs. struggles. The women experienced postpartum mood deviations and illness episodes as being particularly poorly timed. Further challenges included balancing self-care and infant mothering, familial relations, and negative experiences with the health and care systems. Illness acceptance with mindfulness of one’s own and the infant’s needs was a primary strategy for wellbeing, which was complemented by the support of one’s partner and family and postpartum treatment.

**Conclusions:**

Our findings propose that without impeding mood deviations and concomitant challenges, infant mothers with BD can enjoy their new motherhood and experience phase-specific growth equally to healthy mothers. On the other hand, moderate to severe mood deviations can adversely impact the experience of the postpartum year and one’s own sense of mothering. Efforts to prevent postpartum mood deviations need to be complemented with interventions that target phase-specific BD challenges and support wellbeing strategies for both the mother and her infant. In summary, women’s needs to function as infant mothers must be considered in the postpartum treatment of BD.

**Supplementary Information:**

The online version contains supplementary material available at 10.1186/s40345-025-00374-x.

## Background

Adapting to motherhood and caring for an infant may be a potentially stressful process for women with bipolar disorder (BD), as they are far more likely to suffer from postpartum illness episodes than women with any other mental disorder (Jones et al. 2014; Munk-Olsen et al. 2006, 2009). Meta-analytical estimations show an overall relapse risk of 37% in the first year postpartum and a risk of 17% for severe episodes, including mania and psychosis (Wesseloo et al. 2016).

Postpartum illness episodes occur in a critical phase of life for both mothers and children. The transition to motherhood is a major and independent developmental stage in a woman’s lifespan, particularly for primiparous women. It poses significant adjustment demands on women; however, it provides opportunities for psychological growth and the transformation of a sense of self (Cohen and Slade 2000; Stern 2020). For the child, infancy is a major phase of rapid neurobehavioural and social–emotional development, on which early experiences can exert a powerful influence (Fox et al. 2010; Tronick 2007). Current evidence indicates that maternal perinatal mental illness is associated with an increased risk of adverse mental health outcomes for the child (Aktar et al. 2019; Stein et al. 2014). In addition, maternal postpartum mental illness has various emotional, relational and practical consequences for fathers (Davey et al. 2006; Holford et al. 2018).

Although there is an increasing body of literature on the perinatal prevalence and medical management of BD (Sharma and Sharma 2017; Yatham et al. 2018), there is a significant shortage of research on women’s experiences of this high-risk period, including their deliberations to promote wellbeing for themselves and their infants. Two studies that investigated considerations on pregnancy and family planning among women with BD are notable exceptions (Dolman et al. 2016; Stevens et al. 2018). A third study explored how women with BD related to their risk of illness relapse, as well as their concerns and preparations regarding becoming mothers (Anke et al. 2019). It was evident across these three studies that there are some key concerns when women consider motherhood with BD. These include a fear of illness relapse, heredity of BD and negative effects of medication on the foetus and breastfeeding. In addition, women revealed distinct worries about the consequences of illness episodes on their mothering, their child and their partner. Altogether, these studies highlighted a need for health services to be well informed about the complex decisions and demands that perinatal women with BD encounter (Anke et al. 2019a; Dolman et al. 2016; Stevens et al. 2018).

Previous studies were conducted in the preconception/pregnancy phase (Dolman et al. 2016; Stevens et al. 2018) and in the pregnancy/newborn phase (Anke et al. 2019). There is a knowledge gap on BD women’s experiences during the postpartum period, when the risk for illness relapse is at the highest and new motherhood is practised. Therefore, our aim was to conduct a study with a combined focus on the occurrence of mood symptoms and the experiences of infant mothers with BD in the first year postpartum^1^. Our research questions were as follows: (1) How do infant mothers with BD experience their first year, and in what ways are these experiences associated with mood deviations? (2) What challenges do they face, and what do they do to promote wellbeing for themselves and their infants?

## Methods

### Design

This study is part of a larger Norwegian prospective study at Vestre Viken Hospital Trust. The overarching aim is to generate knowledge that may inform perinatal mental health services of psychological and social issues that are important to consider when dealing with women with BD and their families. Infant families in which the mother has BD are studied with qualitative and quantitative assessments in pregnancy, at one, three and 12 months postpartum. The assessments comprise individual interviews with the women and their partners, standardised measurements of the women’s mood symptoms, of the infants’ development, and of the mother-infant and father-infant interactions. The purpose is to investigate different areas of early family life in the context of maternal BD.

In the present study, we applied a mixed-methods design. We used a quantitative approach for the standardised assessment of mood symptoms. We chose a qualitative approach to study the subjective experiences of infant mothers with BD.

### Recruitment procedures and participants

The sample for the present study comprised 26 women diagnosed with BD. The inclusion criteria for the larger prospective study were women with a BD I or II diagnosis who were either pregnant or had recently given birth (within three months) and who had a cohabitating partner who was willing to participate. The exclusion criteria were parental substance abuse, multi-childbirth, premature birth at < 35 weeks, or an infant with a known serious medical condition or syndrome. All eligible participants who consented to participate were included.

Recruitment took place between September 2014 and July 2016. The recruitment sites were mental health outpatient clinics and wards, infant mental health teams^2^, community well-baby clinics, and pregnancy care and maternity wards in the southeastern part of Norway. We also informed participants about the study through the website of the National BD Association and at group psychoeducation courses for patients with BD (Skjelstad et al. 2015). The women’s clinical BD diagnosis was verified from their specialist mental health records and/or by contacting their specialist mental health professional. In addition, the BD diagnosis was assessed with a semi-structured interview and discussed with the research team when needed. For more details about the recruitment procedures, see (Anke et al. 2019).

### Data collection

#### Sample characteristics

Data on demographic and clinical characteristics (including medication and use of mental health services) were collected via questionnaires and interviews at the time of inclusion in the larger study and were updated if needed at 1, 3 and 12 months (Table 1).

Data on infant feeding, breast or formula, were collected at 1 or 3 months (depending on timepoint for inclusion) and updated at 3 and 12 months.

#### Mood symptoms

Data on the presence of mood symptoms were collected at 3 and 12 months with the following instruments:

##### Inventory of depressive symptomatology (IDS)

Depressive symptoms were assessed with the IDS (Rush et al. 1996), a scoring tool for measuring the severity and number of depressive symptoms. It contains 30 items regarding depressive symptomatology and common additional symptoms (anxiety, irritability). The IDS was administered as a clinical interview of approximately 30 min.

##### Young mania rating scale (YMRS)

The YMRS (Young et al. 1978) was used to assess hypomanic/manic symptoms. Symptom scores are based on the person’s reports on 11 items during an interview combined with clinical observation in the situation. The administration time was 10–15 min.

Assessment with the IDS and YMRS is not sufficient to diagnose the event of a mood episode, but it measures the women’s mood status in terms of depressive symptoms in the foregoing week and hypomanic/manic symptoms in the foregoing two days.

In addition to these discrete assessments at two time points, the women’s mood conditions and deviations in the first year were complementarily examined in the study interview (see below).

#### Experiences of infant mothers with BD in the first year

The women were interviewed individually about their experiences by the first author at 12 months, either at the participant’s home (92%) or at Vestre Viken Hospital Trust, as chosen by each woman. The interviews were semi-structured, covering the following postpartum themes: (1) overall experience and perception, (2) the presence and absence of mood deviations and (3) being an infant mother with BD. The questions were posed in an order that was adapted to the natural development of each interview and in an open-ended manner with follow-up questions. When additional topics and discussions were initiated by the participants, they were also explored. On average, the interviews lasted 30 min. The audio of the interviews was recorded and transcribed.

Additional sources for the qualitative dataset on the experiences of the women included the following:

- The IDS and YMRS were administered as clinical interviews, and the women’s elaborations on the assessment items were written down.
- The data were collected during personal meetings lasting 1–2 h, mainly as home visits. It was natural that the women spontaneously spoke about experiences on these occasions. Such accounts were written down.

### Data analysis

#### Mood symptoms

The IDS and YMRS data were categorised descriptively, due to too small subgroups for a statistical comparison of symptom severity.

#### Experiences of infant mothers with BD in the first year

We used inductive thematic analysis to analyse the qualitative data (Braun and Clarke 2006, 2013). The purpose was to systematically identify central recurrent themes in the data linked to the research topic.

To familiarise ourselves with the content and take initial notes, the first author listened to the interviews and read the transcripts of the qualitative dataset repeatedly. With the research questions in mind and paying attention to salient descriptive and linguistic features in the text segments, initial codes were generated case by case. Thereafter, codes were grouped to form candidate themes and subthemes based on patterns and similarities among the different codes. Codes and candidate themes were created and modified in a back-and-forth movement in rereading the data before distinct themes were named and organised hierarchically. The purpose was to identify themes based on prevalence and the ability to depict what is important considering the research questions. Finally, the material was analysed across the cases with the purpose of identifying similarities and variations in experiences among the participants.

Interpretations and identification of themes were reviewed and discussed several times between the authors for revision and refinement and to ensure the validity of the analysis.

The qualitative software NVivo was used as a coding and organising tool.

##### Position of the researchers

Both researchers are clinical psychologists with doctoral degrees (PhDs) in bipolar disorder and with experience in both qualitative and quantitative research. While the first author has her expertise in perinatal and infant mental health, the second author has his expertise in adult mental health and disorders and thus has different but complementary expertise within the field of study.

## Results

### Sample characteristics

Table 1 shows the demographic and clinical characteristics of the sample. All women lived with the infants’ biological father.

**Table 1:** Demographic and clinical characteristics of the sample

Variables	Total *N* = 26	
*Age at inclusion*	M 30.5; range 22–37	
*Satisfaction with life conditions* ^*1*^	M 4.2; range 3–5	
*Years with BD diagnosis*	M 6.5; range 0–16	
	***n***	**%**
*Completed education*		
Primary school	8	31
Secondary school	5	19
Bachelor’s degree	11	42
Master’s degree	2	8
*Employment status when not on maternity leave*		
Working full-time	12	46
Working part-time +/- receiving benefits	4	15
Receiving benefits only	8	31
Unemployed	1	4
Studying (College)	1	4
*Primary Diagnosis*		
BD I	7	27
BD II	19	73
*Number of hospitalisations for BD episodes prior to pregnancy*		
0	10	38
1–3	10	38
≥ 4	6	23
*Mood deviations in pregnancy or within 1 year prior to pregnancy*	11	42
*Satisfactory availability of familial support*		
Partner and grandparents	11	42
Only partner	6	23
Only grandparents	3	12
None	6	23

^1^ Question in first interview in the larger study: “How satisfied are you with your current life conditions– regarding housing, economy and such?” Answers on a 5-point Likert scale: 1 = Dissatisfied, 2 = Not especially satisfied, 3 = Moderately satisfied, 4 = Satisfied, 5 = Very satisfied

Sixty-five percent of the women (*n* = 17/26) initiated breastfeeding at birth and 46% (*n* = 12/26) continued to breastfeed until at least six months. Breastfeeding was mostly chosen by the women who were medication free (*n* = 7/8) or on Lamotrigine monotherapy (*n* = 6/6) at birth. In addition, two women breastfed with antipsychotic medication (one on monotherapy, and one on polytherapy with antidepressants), and two women breastfed with Lamotrigine polytherapy with antidepressants. 35% of the women (*n* = 9/26) did not initiate breastfeeding, mainly due to Lithium medication (*n* = 6/6). Most of these women (*n* = 4/6) expressed sadness over the decision, as it conflicted with their desire to breastfeed. Moreover, an intention to secure sleep was a reason for three women not to breastfeed (*n* = 3). These women seemed to be content with their decision. The prioritising of sleep was also a common reason for women to combine breastfeeding and formula, or to cease breastfeeding after some few months.

### Mood symptoms in the first year

In Table 2, the sample is divided into four subgroups based on symptom severity at 3 and 12 months. The table also displays each participant’s BD type, type and duration of use of main medication, type and duration of use of specialist mental health services, and the women’s main perception of the first year.

**Table 2:** Participants’ BD type, symptom severity at 3 and 12 months, main BD medication, use of specialist mental health services and main perception of the first year

Subgroup	Participant	3 months	12 months	Main BD medication postpartum. Monotherapy or polytherapy	Duration of medication. From pregnancy (P) or months postpartum (pp) to months postpartum (pp)	Total amount receiving medication in each subgroup	Specialist mental health services postpartum. Adult and/or infant mental health	Duration of specialist mental health services. From pregnancy (P) or months postpartum (pp) to months postpartum (pp)	Total amount receiving specialist mental health services in each subgroup	Main perception of the first year postpartum
1. Euthymic^1^ or mild^2^ symptoms	1 BD I	Euthymic	Euthymic	Olanzapine mono	P– 12 pp	8/11, 73%	Adult + infant	P– 1 pp	6/11, 54%	Positive
	2 BD I	Euthymic	Euthymic	Lithium poly	P– 12 pp		Adult	P– 1 pp		Positive
	3 BD II	Euthymic	Euthymic	Lamotrigine mono	P– 11 pp		None	-		Mixed
	4 BD II	Euthymic	Euthymic	Lamotrigine mono	P– 12 pp		None	-		Positive
	5 BD II	Euthymic	Mild-Dep	None	-		None	-		Positive
	6 BD II	Euthymic	Mild-Dep	Lamotrigine mono	P– 12 pp		Adult	P– 3 pp		Positive
	7 BD I	Mixed^5^	Euthymic	Lamotrigine mono	P– 12 pp		None	-		Positive
	8 BD II	Mild-Dep	Euthymic	None	-		Adult	P– 11 pp		Positive
	9 BD II	Mild-Dep	Euthymic	Lamotrigine poly	P– 12 pp		Adult	P– 3 pp		Positive
	10 BD II	Mild-Dep	Euthymic	Lamotrigine poly	P– 12 pp		None	-		Positive
	11 BD II	Mild-Dep	Mild-Dep	None	-		Infant	3 pp– 12 pp		Mixed
2. At least one time point with moderate^3^ affective symptoms	12 BD II	Euthymic	Mod-Dep	None	-	4/6, 67%	None	-	4/6, 67%	Mixed
	13 BD II	Euthymic	Mod-Dep	Lamotrigine poly	P– 12 pp		Adult + infant	P– 3 pp		Positive
	14 BD II	Mod-Dep	Mild-Dep	Lamotrigine poly	P– 12 pp		None	-		Mixed
	15 BD II	Mod-Dep	Mod-Dep	None	-		Adult + Infant	P– 12 pp 1 pp– 12 pp		Mixed
	16 BD II	Mod-Dep	Mod-Dep	Risperidone poly	P– 12 pp		Adult	P– 12 pp		Mixed
	17 BD II	Mod-Dep	Mod-Dep	Lamotrigine poly	P– 12 pp		Adult	P– 12 pp		Mixed/Negative
3. At least one time point with severe^4^ affective symptoms	18 BD I	Hypomanic	Sev-Dep	Lithium poly	P– 12 pp	4/6, 67%	None	-	4/6, 67%	Mixed
	19 BD II	Hypomanic	Sev-Dep	Flupendixol poly	3 pp– 12pp		Adult	P– 12 pp		Mixed
	20 BD II	Mild-Dep	Sev-Dep	None	-		Infant	P– 12 pp		Mixed
	21 BD II	Mild-Dep	Sev-Dep	None	-		Infant	P– 3 pp		Mixed
	22 BD II	Sev-Dep	Sev-Dep	Lithium poly	P– 12 pp		Adult	P– 12 pp		Mixed
	23 BD II	Sev-Dep	Sev-Dep	Lamotrigine mono	P– 12 pp		None	-		Mixed
4. Psychosis	24 BD I	Sev-Dep	Psychosis	Lithium poly	P– 10 pp	3/3, 100%	Infant	1 pp– 12 pp	3/3, 100%	Mixed
	25 BD I	Psychosis	Mod-Dep	Lithium mono	P– 12 pp		Adult + Infant	1 pp– 3 pp 10 pp– 12 pp		Mixed
	26 BD I	Psychosis	Psychosis	Lithium poly	1 pp– 12 pp		Adult + Infant	P– 12 pp 1 pp– 3 pp		Mixed

^1^IDS score 0–13; YMRS score 0–7 ^2^IDS score 14–21; YMRS score 8–20 ^3^IDS score 22–30; YMRS score 21–30

^4^IDS score 31–38 YMRS score ≥ 31 ^5^IDS score 14 and YMRS score 11,5

*Subgroup 1*: 42% of the sample (*n* = 11/26) were either euthymic or had only mild mood symptoms at 3 and 12 months. In the interviews, 91% of the women in the subgroup (*n* = 10/11) evaluated these measures as representative of their general mood state during the first year. One woman (no. 7) revealed having had mild to moderate depressive symptoms for approximately one month at eight months.

Altogether, apart from one woman, subgroup 1 was identified as having had a relatively stable first year in terms of mood symptoms.

*Subgroup 2*: 23% of the sample (*n* = 6/26) had moderate mood symptoms of depressive polarity at 3 and/or 12 months. None of the women confirmed having had hypomanic/manic symptoms. The two women (nos. 12 and 13) who were euthymic at three months dated the initiation of their depressive symptoms to eight (no. 12) and ten (no. 13) months. For woman no. 14, the moderate depressive symptoms at three months gradually alleviated to milder symptoms, which persisted at 12 months.

The three women (no. 15, 16 and 17) who had moderate symptoms at both time points described recurring struggles with depressive symptoms over the first year.

Overall, subgroup 2 was identified as a group with moderate depressive symptoms of various lengths during the first year.

*Subgroup 3*: Another 23% of the sample (*n* = 6/26) had severe mood symptoms of depressive polarity at 3 and/or 12 months. Two women (no. 18 and 19) had hypomanic symptoms at three months, and in all, 83% of the subgroup (*n* = 5/6; all women except no. 21) confirmed mood shifts of both polarities during the first year in their interviews.

Overall, subgroup 3 was identified as a group with severe depressive symptoms of various lengths during the first year.

*Subgroup 4*: The last subgroup included three women (12%) who had postpartum psychosis of mixed^3^ (no. 24), depressive (no. 25) and manic (no. 26) characteristics and was identified as a group with a heavy illness load throughout the first year.

See Supplementary file 1 for more information on the participants’ postpartum medication, and Supplementary file 2 for more information on the distribution of the participants’ scores at 3 and 12 months on extracted IDS items and on YMRS.

### Thematic results

**Fig. 1:**
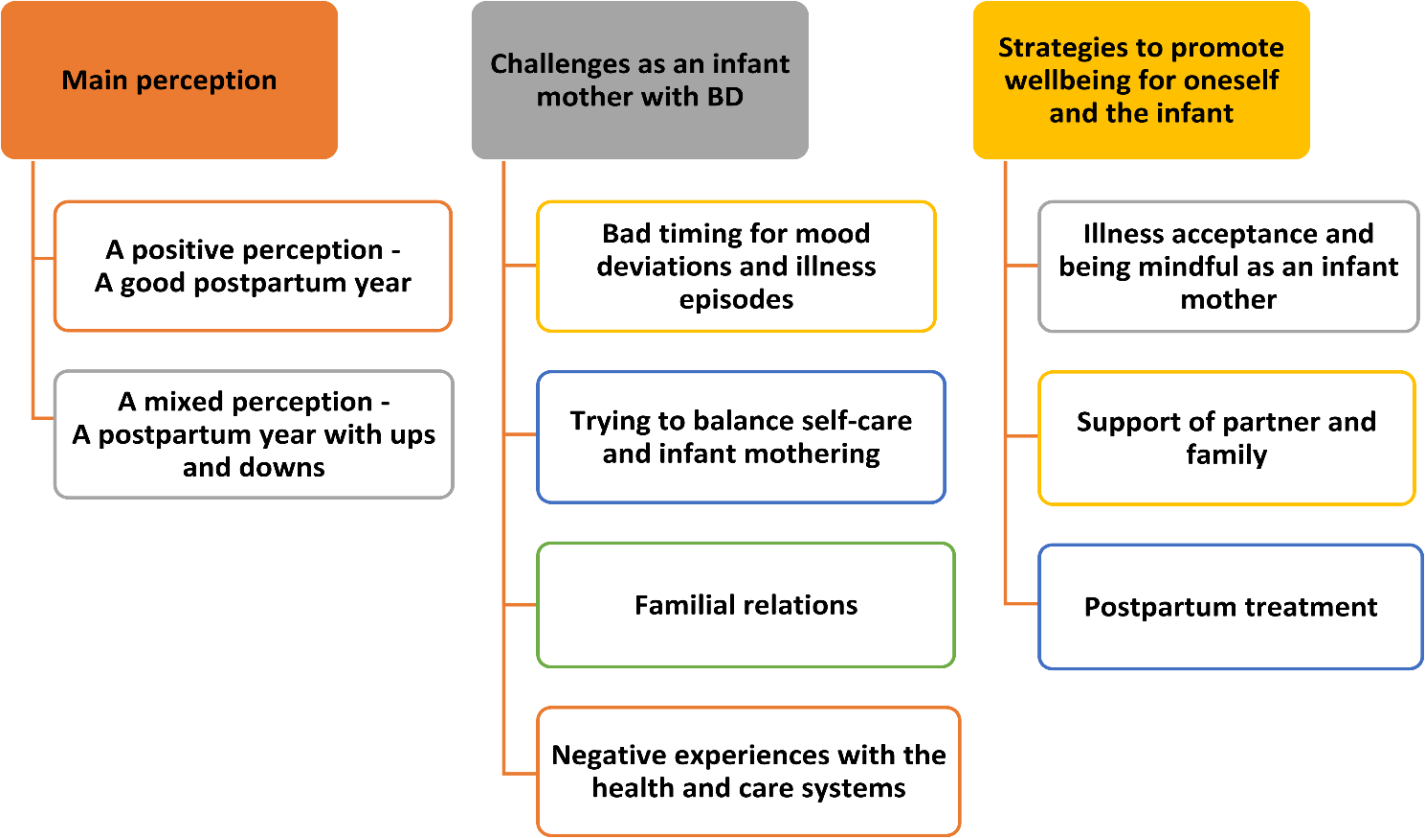
Overview of main themes

Our research questions yielded an overarching thematic structure for the infant mothers’ experiences of the first year, with nine associated main themes (Fig. 1):

Each main theme contains between none and four subthemes, as reported below. We present representative quotes to illustrate the key qualities of each theme and to demonstrate how the themes were grounded in the data.

### Main perception

#### A positive perception—A good postpartum year

38% (*n* = 10/26) of the women reported a main perception of the first year as a positive and good experience and were defined as a positive group (Pos. Gr). Six of the ten women described the experience as better than expected. The women used phrases such as *“It has been a very nice experience.”* (no. 1) and *“It has come out much better than I feared.”* (no. 9).

The main storyline in a large majority of the women’s narratives centred on an experience of positive development and mastery. Primiparous women accentuated a sense of growth and enrichment in accomplishing the role as a new mother: *“It has been a lot of new learning and new values*,* as a mother. I am very positively surprised that it has gone so well to have a baby.”* (no. 13) Most multiparous women (*n* = 3/4) described a sense of reparation in relation to previous tough postpartum experiences: *“In all*,* it has been a ‘boom’*,* because after my first birth I had a postpartum psychosis. This time it didn’t happen*,* so now I feel that it has been normal. The way it should be.”* (no. 2).

As a group, the women gave voice to a pleasure in having become a mother, enjoying their infant and having a daily life that they viewed as normal and stable: *“It gives pleasure to have a child. My priority has been a quiet daily life.”* (no. 1), and *“It has all fallen into place.”* (no. 10).

90% (9/10) of the women with a positive perception were either euthymic or had only mild mood symptoms during the first year (see Table 2). One woman (no. 13) deviated from this pattern and developed moderate depressive symptoms at ten months, as described in the previous section.

#### A mixed perception—A postpartum year with ups and downs

62% (*n* = 16/26) of the sample reported a mixed perception of the first year, defined as apparent positive and negative accounts by each woman. This group was defined as a mixed group (Mix. Gr), although one woman tilted towards a mainly negative perception (no. 17) (see Table 2). The women’s descriptions included phrases such as *“Actually*,* it has varied*,* with a lot of ups and downs. It has been everything: times with deep depression*,* times when I and my husband have struggled*,* and times when it has been really nice.”* (no. 20).

When talking about their negative experiences, mood deviations and the consequences of having been ill were at the forefront for most women: *“It has been heavy. Being on maternity leave was tough. I had a period with heavy depression and then the depression went somewhat up and down.”* (no. 17).

Echoing the Pos. Gr, most women in the Mix. Gr described the value of having become a mother, a (bigger) family and a joy over the infant as dominant positive experiences: *“It’s a joy that we have him* (i.e., the infant). *He is a nice little boy*,* and we enjoy being with him together as a family.”* (no. 26) However, contrasting the Pos. Gr, only a minority of the women in the Mix. Gr (25%, *n* = 4/16) reported a sense of growth and mastery as a mother. These women revealed that the stresses of new motherhood together with episodes of illness made for a difficult process. *“I would have done it again. Yeah*,* actually. Many think it’s strange*,* since I have been so ill. But it has been very good and developing for me.”* (no. 25).

88% (14/16) of the women with a mixed perception of the first year had moderate or severe mood symptoms at 3 and/or 12 months, including postpartum psychosis (see Table 2).

### Challenges as an infant mother with BD

#### Bad timing for mood deviations and illness episodes

Women in both the positive and mixed groups gave accounts of how mood deviations can be significant postpartum challenges. However, the portion of women conveying this was higher in the Mix. Gr (94%, *n* = 15/16) than in the Pos. Gr (30%, *n* = 3/10), and their accounts were more salient and richer in detail, based on their prominent experiences of mood deviations after childbirth.

The women’s narratives revealed how mood deviations are experienced differently and as being particularly unwelcome when being an infant mother. For instance, when women are euthymic during pregnancy, a postpartum relapse can be perceived as especially harsh: *“The pregnancy was so nice. I have never been so well from my illness as when I was pregnant. And then*,* it’s so dramatic and heavy to be ill afterwards. Quite simply shit!”* (no. 22, Mix. Gr).

In more than one-third of the sample (Pos. Gr: 10%, *n* = 1/10 vs. Mix. Gr: 50%, *n* = 8/16), the narratives implied that mood deviations had a negative impact on the women’s sense of being a mother and their mothering confidence: *“I felt that being a mother was two*,* or even four*,* sizes too big for me. (…) I just couldn’t understand what that ‘little thing’ wanted*,* what she needed and what she expected from me. (…) And I can still feel as if I am acting.”* (no. 15, Mix. Gr).

At its extreme, postpartum episodes could be felt as “too much”, causing feelings of regret in a few women: *“I have felt it as a really bad idea to have children. Some days*,* I just want to leave. I want to be alone. I cannot take being with them. Nothing I do works.”* (no. 17, Mix. Gr). Even suicidal ideation was triggered in a small minority of three women (12%) during severe depression and near-psychotic states. *“I have had thoughts of suicide when in deep depression. But it’s no longer a choice*,* because of her* (i.e., the infant).*”* (no. 20, Mix. Gr).

##### Fearing postpartum mood deviations

The narratives revealed a recurring fear of becoming ill related to “bad timing”. Notably, such accounts were more common in the Pos. Gr (60%, *n* = 6/10) than in the Mix. Gr (19%, *n* = 3/16). The fear could either be provoked by symptoms, *“I had hallucinations a couple of times*,* and got really afraid that I might develop a depressive psychosis.”* (no. 16, Mix. Gr), or by situations commonly viewed as symptom triggers, *“When I get tired*,* I have this fear of becoming depressed. Then*,* all the time*,* there is this fear of ‘What’s happening?’*,* ‘Is this the start of becoming ill?’. These worries are an extra burden. (…) I am so afraid of becoming ill!”* (no.9, Pos. Gr). A taxing loop of fear, vigilance and stress was disclosed by some of the women.

##### Depressive mood deviations

Of the mood polarities, depressive deviations were the most common, with 54% (*n* = 14/26) of the women having IDS scores at 3 and/or 12 months in the moderate to severe range (Table 2). 62% of the women (Pos. Gr: 40%, *n* = 4/10 vs. Mix. Gr: 75%, *n* = 12/16) described how depressive mood deviations may impact their mothering. A recurring feature was a sense of something missing, an absence of something that should have “been there” in their mothering. This included a sense of not being mentally present and available for the infant: *“Sometimes I must pull myself together to be present with him. Because I am gone. In my mind.”* (no. 13, Pos. Gr). Furthermore, women described a lack of energy in their mothering: *“It was all quite heavy. I didn’t have the energy for almost anything. It was not possible for me to be alone with him all day*,* to bear all the responsibility.”* (no. 17, Mix. Gr), as well as a lack of patience, *“This time*,* when I was deep down*,* I couldn’t handle it. For example*,* when she was whiny and couldn’t fall asleep. I didn’t handle it the way I usually do*,* with patience*,* like nowadays.”* (no. 7, Pos. Gr). Some women experienced challenges as even more severe as the infants grew older. Coping with depression while caring for an infant who was more awake during the day, interactive and mobile could be considered incompatible endeavours: *“I don’t think I would have managed to have him at home the whole day now. I would have become even more depressed. It went well when he was little. But I feel that if he demands too much of me*,* if I must*,* you know*,* pay attention all the time*,* then it gets too much for me.”* (no. 23, Mix. Gr).

##### Hypomanic mood deviations

Women with hypomanic symptoms (Mix. Gr: 31%, *n* = 5/16) talked about irritability, agitation, racing thoughts and “being carried away” in different activities as particular mothering challenges. One woman described the ironic nature of her absorption in baby accessories on the internet while missing out on her infant’s bids: *“When I was hypomanic-manic*,* I began with all these baby-accessories on the internet. (…) I got all caught up. I bought a lot of things and spent a lot of time on the net. You are in these chat-groups*,* and it’s really easy to get hooked. But then I found out that ‘Hey*,* this is wrong!’. I kind of buy all these nice baby-things and chat with others about baby-stuff*,* while my baby is crying beside me. And I don’t notice! That’s not the point! I should be with my baby!”* (no. 19, Mix. Gr).

##### Postpartum psychosis

Being ill with postpartum psychosis was described as an overwhelming experience (Mix. Gr: 19%, *n* = 3/16), which profoundly contrasted with women’s prenatal expectations of new motherhood. A deep concern for the impact on the mother-infant relationship was evident, intensified by the separation when being hospitalised: *“The worst*,* it’s almost like I cannot forgive*,* is that I felt it as if they robbed my son away from me. (…) To see them* (i.e., father and son) *leaving me*,* ruined me. Time after time. I held*,* I just held my son close to me the whole time when they were present. Just held.”* (no. 25, Mix. Gr).

In addition to the acute phase of psychosis being highly stressful, the recovery phase represented more drawn-out maternal challenges in terms of repairing the bonding with the infant, processing the grief of what had been lost, and dealing with worries: *“It took me at least six months before I felt that ‘This is ok.’ (…) I was so worn out of everyone telling me about ‘Attachment*,* and mother*,* mother*,* mother.’ ‘That he will be damaged*,* get ADHD*,* be totally crazy.’ I thought*,* ‘Oh*,* my God*,* with his BD genes! How will everything end?’ (…) My husband has ensured me that ‘You have had your maternal instinct all the time.’ And I do feel that I have had it. To protect him*,* feed him*,* give him dry diapers. But the maternal love*,* to feel that he is mine and that I love him. It wasn’t there*,* I couldn’t feel it.”* (no. 25, Mix. Gr).

#### Trying to balance self-care and infant mothering

A large group of women (Pos. Gr: 50%, *n* = 5/10 vs. Mix. Gr: 81%, *n* = 13/16) spoke about the challenges in trying to maintain self-care and preventive measures while practising mothering and attending to the infant’s needs: *“It’s more difficult now*,* because you have someone else to think about. He takes a lot of my energy*,* or it’s more like you want to give him what you have. (…) Anyway*,* it’s difficult to ‘refuel’*,* because you give what you have during daytime*,* or even at nighttime. When he needs it.”* (no. 12, Mix. Gr).

The main challenge was to secure sleep—night awakenings and possible sleep deprivation are inherent in caring for an infant: *“I had to get up every second to third hour* (i.e., to feed the infant). *I had too little sleep*,* and you know*,* that can result in me becoming hypomanic.”* (no. 18, Mix. Gr) Trying to avoid exhaustion and stress was also difficult, *“I am afraid of getting exhausted*,* because I know that then I am more vulnerable. But*,* you know*,* the first year with a baby*,* you get exhausted.”* (no. 6, Pos. Gr).

Some women on medication found that side effects could negatively affect their ability to mother: *“I think it’s difficult to be on medication. So many of the medicines make me lethargic or make me sleep too tight. And it can’t be that way when I have a baby. So*,* actually*,* now I don’t use them* (i.e., her prescribed medication).*”* (no. 16, Mix. Gr).

#### Familial relations

Challenges in their familial relationships (i.e., infant, partner and grandparents) were most prominent in the reports of women in the Mix. Gr (75%, *n* = 12/16), who often disclosed challenges in at least two types of familial relationships. Familial challenges were less common in the Pos. Gr (40%, *n* = 4/10) and were mainly associated with one type of relationship. The common subject in both groups was concern about the familial impact of postpartum mood deviations: *“When I become depressed it’s like it ‘contaminates’ the others around me. The total lack of energy and the mood swings affect everybody.”* (no. 20, Mix. Gr).

Moreover, there were various challenges and concerns related to different family members.

##### The infant

Even though the women explicitly talked about mood deviations affecting their mothering, only a minority of the women (Pos. Gr: 20%, *n* = 2/10 vs. Mix. Gr: 25%, *n* = 4/16) acknowledged that their infant had been subjected to overt mood symptoms, such as the mother’s apparent anger or sadness. This was troubling for the women to think about, *“He has been present in situations where I haven’t been stable. There has been shouting and yelling*,* or*,* like hard negative talk. So*,* he has noticed*,* and I think it’s like ‘What happens now?’ for him*,* because he isn’t used to it. (…) It’s not alright. No*,* it isn’t. So*,* you try to avoid such situations.”* (no. 23, Mix. Gr).

##### The partner

Half of the women in both groups (Pos. Gr: 50%, *n* = 5/10 and Mix. Gr: 50%, *n* = 8/16) highlighted the partner as the main person affected by their BD postpartum. This comprised the women’s need for support in euthymic phases (i.e., to maintain self-care measures) and aid in illness phases (i.e., partner taking over care responsibilities for the infant). Typically, women in both groups saw this as a challenge—he had to be prepared to make more effort than what was “his share” as a new parent: *“He takes a lot of responsibility*,* even at nights. Also*,* other things to relieve me*,* so he does a lot. Does the cooking*,* fixes and arranges. He really does everything.”* (no. 13, Pos. Gr).

The narratives also revealed strained and conflicted couple relationships in the first year, mainly in the Mix. Gr (Pos. Gr: 10%, *n* = 1/10 vs. Mix. Gr: 50%, *n* = 8/16). A common reason was the stress inflicted by episodes of illness and the woman’s altered behaviour: *“We have had rough times*,* but most of the time it has been good. (…) A couple of times it has been because of money. It comes easy for me to spend a lot of money*,* and not to think over it.* (i.e., when ill in hypomania)” (no. 19, Mix. Gr).

##### The grandparents

The main challenge in relationships with grandparents was that they were not available or supportive when needed (Pos. Gr: 20%, *n* = 2/10 vs. Mix. Gr: 44%, *n* = 7/16). Some women reported sadness when grandparents lived far away but expressed it as a straightforward and manageable feeling. A more complex and disappointed feeling was displayed when the lack of support was either contrary to promises given during pregnancy or because of intergenerational conflicts: *“It’s like my parents drain me of energy. (…) It has to do with our family history. (…) It’s not good for me to have to depend on my mother.”* (no. 15, Mix. Gr). Notably, for some women, the transition to motherhood could reactivate troubled childhood memories of having lived with a parent with BD or other mental disorders and complicate the current parent–grandparent relationship: *“My father is rather ill from time to time* (i.e., BD). *(…) It was an unstable family situation*,* without safety. We never knew what the next day would be like. (…) After I had my own children*,* memories have returned and become stronger. So*,* my relationship with my mother and father has become more complicated.”* (no. 16, Mix. Gr).

#### Negative experiences with the health and care systems

Unfortunately, instead of feeling supported and helped, about a quarter of the women (Pos. Gr: 30%, *n* = 3/10 vs. Mix. Gr: 25%, *n* = 4/16) disclosed unwanted negative experiences with the health and care systems. Women did not report dissatisfaction with professionals’ medical or psychiatric knowledge. Rather, they described experiences of feeling denigrated and met with prejudiced assumptions as infant mothers with BD. For instance, when care information was presented normatively without individual adaptation, it could cause a feeling of having failed and not belonging “as a mother amongst mothers”, *“I had to quit attending these ‘mother-baby-groups’ and the community well-baby clinic. And I avoided the internet. Well*,* just everyone who said that ‘the mother is the most important’ (…) and declared proper daycare*,* etcetera. I have had help and babysitters since he was three weeks old! Not because I wanted*,* but because it was urgent and vital! And the focus on breastfeeding and the immune system. We have given him infant formula from day one! Everything that I felt I had done wrong was only confirmed.”* (no. 25, Mix. Gr).

Furthermore, encounters with the care systems could be considered suspicious surveillance, resulting in remorse for having reached out for help: *“They came from the child protection services and observed me. I needed help. They said that the children had to be shielded. I was stunned by their language because people in child protection have power. So*,* I wondered what their solutions were. They meant that I had to contact people in my network*,* that family and friends had to come and help me. I said that ‘I don’t have anyone to step up 24 hours a day!’ (…) So*,* they observed and observed and concluded that they had to come back and observe. (…) It was a very tough situation*,* I really must say.”* (no. 21, Mix. Gr).

Notably, based on their experiences, some of the women revealed regret about having disclosed their BD to health professionals: *“I didn’t think so much about it in the beginning. But now*,* when I feel like I am myself again*,* it’s a label that isn’t so easy to relate to. (…) In the beginning it was important for me to be open. And I regret that now. (…) For instance*,* at the well-baby clinic*,* I feel that I must accept assumptions about my situation*,* when I actually want to protest: ‘But honestly*,* it isn’t like that.’ Things that are tough to hear when you are a new mother and want to be safe and good for your baby. (…) In a way*,* it’s kind of humiliating. So*,* I think that having disclosed is what I regret the most.”* (no. 6, Pos. Gr).

### Strategies to promote wellbeing for oneself and the infant

#### Illness acceptance and being mindful as an infant mother

A personal acceptance of “how things are” when having a recurring mental illness and being an infant mother seemed to be a primary process for health-promoting priorities to become integral in postpartum daily life and to include both the mother and the infant. All women in the Pos. Gr (*n* = 10/10) provided explicit reports on trying to be mindful of their illness and their daily priorities for their own and their infant’s wellbeing and needs. The same was evident for 75% (*n* = 12/16) of the Mix. Gr, *“I have become more conscious about different things. I think I have accepted and reconciled with my illness in another way now. (…) All has become calmer and more natural. I take more precautions. For instance*,* no computer or screen devices before bedtime. And I am better at planning ahead*,* to avoid stress. Before the baptism*,* I made a list. I think and organise ahead*,* so I don’t have to do everything at the same time. And I am better at ‘leaving things at that’. Before*,* I didn’t want to relate to the illness*,* I denied and trivialised. And it went bad.”* (no. 2, Pos. Gr).

Despite the inherent challenges in practising self-care as an infant mother, several women spoke about a maternal responsibility to try to stay well. Keeping their infant in mind added motivation to health-promoting priorities: *“You must be aware of your own limitations and think ‘Now it’s not only about me.’ I must take extra good care of me*,* to take care of my baby. I must have consideration for her wellbeing. Therefore*,* I make sure that I sleep and eat well*,* you know*,* live healthy.”* (no. 1, Pos. Gr) What health-promoting priorities each woman was mindful of could differ based on individual needs. For instance, *“I am very sensitive for stress. I need to prioritise. What appointments can I cancel? What can be downgraded and be more basic?”* (no. 6, Pos. Gr).

Being mindful of the infant’s wellbeing included an awareness of one’s own impact on the infant: *“I think I am very conscious of how I behave with him* (i.e., the infant). *That’s why I tell my husband or my mother-in- law when I have a bad day. They assist so that I am not alone with him.”* (no. 16, Mix. Gr) This also implied prioritising energy to mothering, *“It is much easier now*,* when he is at day care. I can rest and be alone during the day*,* and then I have the energy to give care in the afternoon and evening.”* (no. 17, Mix. Gr).

#### Support of partner and family

In the previous section on challenges, we described women’s concerns about the impact of postpartum illness on family members. These concerns were largely associated with the fact that a majority of the sample (Pos. Gr: 70%, *n* = 7/10 vs. Mix. Gr: 75%, *n* = 12/16), viewed familial aid as almost essential for wellbeing and to manage mothering in the first year.

The most reported support from the partner was of a practical nature, *“I was very deep down*,* so he* (i.e., partner) *had to take over the maternity leave and take care of our daughter a lot. They were outdoors most of the days. It helped and was really important*,* we couldn’t have managed otherwise.”* (no. 20, Mix. Gr). The partner’s assistance, allowing the mother to sleep, was precious: “*You know what they say*,* ‘Infant parents don’t sleep at night.’. Well*,* I do*,* but my husband doesn’t. He takes the night shifts.”* (no. 10, Pos. Gr).

Furthermore, the partner’s emotional support was highly valued: *“In that period* (i.e., when depressed), *my husband emphasised*,* ‘Take one day at a time.’*,* ‘Don’t think about what will happen tomorrow.’*,* ‘Set up small goals for today*,* and then you handle tomorrow when tomorrow comes.’”* (no. 9, Pos. Gr).

The way in which partner support was initiated varied within the sample. Some couples appeared to have talked about the forthcoming challenges and preventive measures during the pregnancy. In other couples, the woman had to be explicit on urgent needs for aid: *“Well*,* I had to tell him sometimes that ‘You must take him this night. You really must*,* even if you are going to work in the morning.’. I don’t think he was so observant of how bad it was for me.*” (no. 12, Mix. Gr). Alternatively, a health professional had to address the matter: “*We were at the psychologist’s office*,* and she told him*,* ‘This doesn’t work*,* you must take more part in this. Even if you work*,* you must assist.’”* (no. 15, Mix. Gr).

Although the partner’s support was primary among the women, in cases where grandparents were available for aid, it was vital, *“Our family has been here. Since I haven’t been present neither physically nor mentally*,* it has been a huge comfort to know that my baby has been taken well care of. And my husband has received assistance from our parents.*” (no. 25, Mix. Gr) Notably, when having received helpful support, there could be hints of reparation in familial relations that previously had been conflicted: *“When I was little*,* I only wanted to be with my father. (…) I felt much safer with him. Now my mother has been baby-sitting several times and it has gone well. I have given her clear directions about things. There are so many things that I feel she did wrong in my childhood. (…) I have talked with her a lot about it*,* and I think she does it well now.”* (no. 7, Pos. Gr).

#### Postpartum treatment

Although some women voiced challenges in dealing with the health and care systems, almost everyone (92%, *n* = 24/26) felt that they needed some kind of postpartum treatment, preferably at the specialist mental health care level. 31% of the sample (Pos. Gr: 40%, *n* = 4/10 vs. Mix. Gr: 25%, *n* = 4/16) felt satisfied and safe with pharmacotherapy only, *“I have been very confident that my medication is effective*,* so I haven’t been afraid* (i.e., of illness relapse).*”* (No. 2, Pos. Gr).

Instead of medication, 19% of the sample (Pos. Gr: 10%, *n* = 1/10 vs. Mix. Gr: 25%, *n* = 4/16) preferred psychotherapy, either individually or with an infant mental health team, *“It has been very useful with help from the infant-mental health team. To talk about our experiences*,* without being viewed as terrible parents.”* (no. 15, Mix. Gr).

Finally, several women favoured a combination of medication and psychotherapy (Pos. Gr: 40%, *n* = 4/10 vs. Mix. Gr: 44%, *n* = 7/16), *“I felt that with both measures - more frequent therapy*,* starting medication - we retrieved it in a way*,* and I avoided to touch the bottom. The access to someone to talk to is very important for me. It helps me to reach an inner calm. (…) I need both.”* (no. 9, Pos. Gr).

## Discussion

To the best of our knowledge, the current mixed-methods study is the first to report on mood symptoms together with perceptions, challenges and strategies to promote wellbeing among infant mothers with BD in the first year postpartum, a high-risk period of illness relapse. In the following, we discuss the main findings.

### Mood symptoms and deviations in the first year

Whereas 42% of the women were assessed to be euthymic or to have only mild mood symptoms at 3 and 12 months, 58% showed moderate to severe symptoms at either or both time points. Since our symptom data are not sufficient to diagnose the event of a mood episode, a direct comparison with other studies on BD postpartum relapse is not possible. However, the women’s reports revealed that in most cases, the 3- and 12-month assessments validly represented their illness burden during the first year, indicating BD recurrence in approximately half of the sample. Most recurrences were depressive, corresponding with other postpartum studies (Driscoll et al. 2017; Wesseloo et al. 2016), although there were cases of hypomania/mania and psychosis as well.

73% of the sample received pharmacotherapy concordant with recommendations for prophylactic treatment (Yatham et al. 2018), with the large majority using medication throughout pregnancy to 12 months. Given that prophylactic pharmacotherapy decreases postpartum relapse in comparison with no medication (Wesseloo et al. 2016), the symptom burden in the sample needs consideration.

Most studies on postpartum prophylactic pharmacotherapy have investigated lithium, whereas the corresponding effects of lamotrigine and antipsychotics have scarcely been studied (Wesseloo et al. 2016). One exception is a study in which mood stabilisation during pregnancy with lamotrigine was compared with lithium for the prevention of severe BD episodes requiring hospitalisation within three months postpartum. There was no significant difference in the risk of postpartum hospitalisation between women who used lamotrigine and those who used lithium, suggesting that lamotrigine may be a reasonable BD treatment for pregnant women who are vulnerable to depression (Wesseloo et al. 2017). However, less severe mood episodes and recurrences over a longer postpartum period were not investigated. In the current study, only 23% (*n* = 6/26) of the participants received lithium. Otherwise, lamotrigine and antipsychotics were the main medications in mono- or polytherapy. Among the women who received lithium, only one was euthymic throughout the first year, three were hospitalised for postpartum psychosis, and two had severe depressive symptoms at least once. Thus, unfortunately, the women in the sample who used lithium did not experience the expected protective postpartum effect. Furthermore, 42% of the sample entered pregnancy with a recent or ongoing illness relapse, and there was a high rate of prior hospitalisations (≥ 4) in 23% of the sample. Both conditions are proposed to aggravate the risk of postpartum relapse (Doyle et al. 2012; Viguera et al. 2011). Finally, since a majority of our sample received pharmacotherapy and/or specialist mental health services, they are likely to reflect women with a severe illness course.

Altogether, the above factors may offer some explanation to the divergence between a high proportion of the sample receiving pharmacotherapy and several nonetheless presenting evident mood deviations.

### Main perception of the first year

Having experienced euthymia and/or mild mood symptoms vs. moderate and/or severe mood deviations convincingly differentiated between women who had a positive (38%, Pos. Gr) vs. a mixed (62%, Mix. Gr) perception of the first year. Moderate to severe mood deviations, including psychosis, were identified as major sources of negative experiences in the Mix. Gr.

In addition to the fact that being well or unwell implies different experiences, the women’s perceptions may also be understood within the life phase of new motherhood. Notably, several studies have reported that for many women with severe mental illness, including BD, the ability to cope with mothering has a high priority. Managing mothering entails meaning and value and is viewed as signifying normalcy as opposed to illness suffering and illness identity (Diaz-Caneja and Johnson 2004; Dolman et al. 2016; Montgomery et al. 2006; Nicholson et al. 1998; Tjoflåt and Ramvi 2013). Our findings corroborate the literature since women in both groups emphasised the value of having become a mother and experiencing delight in the infant. However, whereas an experience of positive development and mastery was prominent in the narratives of the women in the Pos. Gr, only a minority of women in the Mix. Gr expressed such postpartum views. In contrast, half of the women in the Mix. Gr revealed strains in their sense of being a mother and in their mothering confidence. This is a notable concern, as experiences in the postpartum period are regarded as crucial in women’s emerging representation of “self-as-mother” (Cohen and Slade 2000; Stern 2020). It is arguable that euthymia and mild mood symptoms (i.e., Pos. Gr) helped the women achieve the expected and desired developmental tasks in new motherhood, whereas moderate and severe mood deviations (i.e., Mix. Gr) complicated such achievements by causing explicit conflicting “dual demands” of illness and motherhood (Ackerson 2003). In addition, the normal stress of infant mothering may have been exacerbated by illness-related issues.

Finally, a psychological process of new role adaptation and integration may be challenged when mood deviations add instability to a transition process that is destabilising in itself (Cohen and Slade 2000; Stern 2020).

### Challenges as an infant mother with BD

The women’s fear of postpartum episodes and their consequences on mothering, the child and partner correspond with the concerns of women with BD when considering pregnancy or being pregnant (Anke et al. 2019a; Dolman et al. 2016; Stevens et al. 2018). However, the dearth of studies on mothering with BD limits our efforts to compare the challenges of infant mothers with those of mothers with older children. Still, three qualitative studies on parenting with BD (entailing 90% mothers vs. 10% fathers) reported parallel experiences of illness relapse affecting parenting abilities, struggles in balancing own and the child’s needs, and fear of being viewed as an inadequate parent (Tjoflåt and Ramvi 2013; Venkataraman and Ackerson 2008; Wilson and Crowe 2009).

Furthermore, in several domains, our findings on challenges as an infant mother converge with previous qualitative research on challenges and distress when living with BD (Warwick et al. 2019). For instance, common challenges include fearing symptoms and illness relapse (Fletcher et al. 2013; Lee Mortensen et al. 2015), experiencing mood deviations and their consequences (Fernandez et al. 2014; Inder et al. 2008), pharmacological side effects affecting daily functioning (Crowe et al. 2012; Inder et al. 2010), efforts required for self-care measures (Fletcher et al. 2013; Jönsson et al. 2008), negative self-view and low self-confidence (Crowe et al. 2012; Inder et al. 2008), distress in close relationships (Granek et al. 2016; Lee Mortensen et al. 2015) and encountering stigmatised assumptions (Crowe et al. 2012; Siegel-Ramsay et al. 2023).

Taken together, our findings are supported by a significant body of research. That is, the challenges seem likely to be generic to having a recurring mental illness such as BD and are fundamental ways in which BD may affect living, whether as an “individual” or in the role of a new mother. However, our present findings extend previous knowledge in showing the impact of these generic challenges on postpartum motherhood.

In line with their weighty impact on the women’s main perception of the postpartum year, mood deviations were viewed as major challenges and as coming with bad timing. Typically, the women felt that depressive deviations could impede mothering through creating a lack of mental presence, energy and patience. These subjective accounts concur with mother–infant interaction data in previous studies on the same sample. At the group level, affective flatness and reduced involvement were significant maternal deviations in mother–infant interactions at 3 and 12 months compared with mother–infant interactions with healthy mothers^4^ (Anke et al. 2019b, 2020).

The women’s descriptions of irritability, agitation and “being carried away” in hypomanic deviations also imply maternal behaviour that may impede positive mother–infant interactions. Indeed, accumulating data indicate difficulties in mother–infant interactions when mothers have BD (Aran et al. 2021; Biaggi et al. 2024; Bjertrup et al. 2022), as well as in maternal postpartum bonding (Boekhorst et al. 2021). These findings are important because the quality of mother–infant interactions is proposed to be an essential mediator between maternal mental illness and child outcomes (Howard and Khalifeh 2020; Stein et al. 2014). Thus, both subjective (i.e., maternal accounts) and objective (i.e., standardised interaction assessments) data indicate a need to focus on potential challenges in mothering and mother–infant interactions in postpartum BD care. This notion is further supported by data on an elevated risk for psychopathology when having been exposed to parental moderate-severe BD relapse between 0 and 2 years, compared to later in childhood (Goodday et al. 2018).

Our inferences on how postpartum psychosis can represent challenges are limited because we obtained data from only three women. The women’s experience of it as a highly stressful event that contrasted with the expectations of new motherhood is consistent with previous literature (Edwards and Timmons 2005; Engqvist et al. 2011; Glover et al. 2014; Heron et al. 2012; Robertson and Lyons 2003). A predominant theme in our sample was the disrupting effect on the mother–infant relationship. In Norway, there are no inpatient units with conjoint admission of mothers and infants in cases of postpartum psychosis (Glangeaud-Freudenthal et al. 2014), causing compulsory mother–infant separation. In essence, our findings are consistent with previous literature on postpartum separation having negative outcomes on the mother–infant bonding process (Johnson 2013).

Feelings of regret for having given birth to a child and for having suicidal ideation are serious challenges to deal with in new motherhood. A small minority of women in our sample disclosed having had these experiences in severe depression and near-psychotic states. 12% reported suicidal ideation, corresponding with the prevalence range of 5–14% reported in other perinatal studies (Orsolini et al. 2016). Although perinatal suicides are rare in the general population, perinatal suicidal ideation is considered a risk factor for suicidal behaviour (Orsolini et al. 2016), and postpartum women with severe depression and psychosis are at particular risk (Johannsen et al. 2016; Khalifeh et al. 2016; Oates 2003). Our findings confirm the importance of attending to complicated maternal feelings as well as assessing suicidal ideation in BD women with severe postpartum episodes to initiate needed support and help.

In view of the evident postpartum challenges, the negative experiences with the health and care systems are troubling. Women described having felt denigrated and met with prejudice from professionals, which resonates with the wider literature on mothers with severe mental illness (Diaz-Caneja and Johnson 2004; Nicholson et al. 1998; Wilson and Crowe 2009). Such experiences can make mothers less likely to seek help for the challenges they encounter. Indeed, we identified remorse of having disclosed their BD diagnosis and having reached out for help. A reluctance to access services when needed in the postpartum period is unfortunate since early targeted interventions may be preventive for subsequent challenges in mother–infant interactions (Anke et al. 2019b, 2020; Aran et al. 2021; Biaggi et al. 2024; Bjertrup et al. 2022) and in mothering (Tjoflåt and Ramvi 2013; Venkataraman and Ackerson 2008; Wilson and Crowe 2009).

Decision-making on breastfeeding may also involve challenges for infant mothers with BD, due to their medication or the dilemma in weighing self-care (e.g. regular sleep) with breastfeeding (Baker et al. 2021; Frayne et al. 2023). Our findings suggest that being explicitly advised against breastfeeding due to medication use (e.g. Lithium) may cause more negative feelings in infant mothers, such as sadness and grief, than a decision to prioritise sleep over breastfeeding. One reason may be more felt autonomy over own breastfeeding decision in the latter situation (Frayne et al. 2023). The portion of breastfeeding women in our sample (65%) is comparable with the results in a recent study from UK, in which 66% of women with BD breastfed (Baker et al. 2021). However, fewer women in our sample breastfed in comparison with the general Norwegian population, at one month, 65% vs. 93%, and at six months, 46% vs. 78% (Myhre et al. 2020).

### Strategies to promote wellbeing for oneself and the infant

The descriptions of accepting “how things are” when having BD and being an infant mother mirror the process described by Tjoflåt and Ramvi (2013) of parents gradually admitting and accepting their BD diagnosis and reaching a realistic view of themselves in a parental role. It is widely claimed that illness acceptance and a “recognition of the problem” are pivotal in a recovery process with BD and in finding strategies for wellbeing (Fernandez et al. 2014; Russell and Browne 2005; Warwick et al. 2019b). Our findings further suggest that illness acceptance prompts the mother’s progress towards mindfulness of determining how the illness may impact her infant. With an illness acceptance and “keeping the baby in mind” (Slade 2002; Slade et al. 2005), the mother can exhibit the responsibilities of motherhood (Nelson 2003) and employ wellbeing strategies not only for herself but also for the infant. For instance, postpartum rest and sleep should be prioritised not only to stay well but also for the sake of energy to support sensitive infant mothering.

The women’s emphasis on the importance of practical aid as well as emotional support has been confirmed across BD studies, e.g. when considering pregnancy and being pregnant (Anke et al. 2019a; Dolman et al. 2016; Stevens et al. 2018), for good parenting (Tjoflåt and Ramvi 2013) and in general life and recovery (Fernandez et al. 2014; Michalak et al. 2006; Warwick et al. 2019b). Building and negotiating a supporting network for oneself and the infant is considered a generic theme in the transition to motherhood, since helpful others can facilitate for the new mother to commit herself to primary mothering tasks (Stern 2020). Our findings suggest this to be fundamental under the circumstances of “dual demands” of illness and motherhood (Ackerson 2003).

Postpartum medication and psychotherapy were individually, or combined, viewed as necessary for a large majority of our sample. The first, as a tool for relapse prevention (Jones et al. 2014; Yatham et al. 2018), and the second as a tool for expressing and better managing postpartum challenges and concerns related to the illness. On the one hand, mood deviations may lead to accumulating strains; on the other hand, such stress may further undermine women’s wellbeing. Thus, it is likely that mood deviations and concomitant challenges may become interwoven in reciprocally reinforcing negative patterns where both need to be targeted in postpartum BD care.

## Clinical implications

Our findings underpin the importance of health and care professionals considering the whole family system in a longitudinal way (Howard and Khalifeh 2020) when delivering care to postpartum women with BD. Professionals need to be sensitive to how BD mood deviations—depressive, hypomanic/manic or psychotic episodes—may impact an infant mother and to translate how common BD challenges may affect in the postpartum context.

Our current findings illustrate that we cannot control the event of postpartum mood deviations even with our best efforts to prevent illness relapse with prophylactic pharmacotherapy. A broader perspective on preventing mood deviations *and* targeting postpartum challenges allows for different clinical ports of entry to reduce suffering for infant mothers with BD and to positively impact their infants and families. Efforts to prevent postpartum mood deviations therefore need to be complemented with interventions that are adapted to phase-specific BD challenges and support wellbeing strategies for both the mother and her infant.

Finally, our findings make a case for thoughtful preconception counselling for women with BD when they consider becoming pregnant. Literature and clinical knowledge indicate that women with BD experience that family, friends and health professionals may discourage them from becoming pregnant, and they may feel doubtful themselves (Dolman et al. 2016; Stevens et al. 2018; Viguera et al. 2002). Indeed, there is a high risk of illness relapse postpartum, including very severe episodes. However, our findings suggest that predictions of postpartum illness burden are difficult, even when it comes to women with a severe BD history. In our study, women with BD I (*n* = 7), with a history of psychoses and the most frequent and/or longest hospitalisations represented opposite extremes of postpartum illness burden. Three women were euthymic or had only mild symptoms, a positive perception of the postpartum year, and experienced growth and enrichment in their motherhood. Four women on the opposite extreme experienced severe depression or psychosis. Still, their perception of the year was mixed, with both negative and positive experiences, and two reported a sense of growth and positive development in despite of their heavy challenges. Family planning is a delicate and personal matter for a woman and her partner to decide together. Access to updated information about the risks and challenges, together with a consciousness of what is needed of themselves and their network to carry out preventive measures, is critical for preconception deliberations and decision making about pregnancy. We hope that the findings of our study may assist women across the range of severity of BD in their considerations together with their partner and their health professional.

## Conclusion

In conclusion, the findings from this study will contribute to informing perinatal mental health services for women with BD and their families, with a particular focus on the first postpartum year.

Our findings propose that without impeding mood deviations and concomitant challenges, infant mothers with BD can enjoy their new motherhood and experience phase-specific growth equally to healthy mothers. On the other hand, moderate to severe mood deviations can adversely impact the experience of the postpartum year and one’s own sense of mothering. Moreover, mood deviations and concomitant challenges may become interwoven in reciprocally reinforcing negative patterns, both of which need to be targeted. In sum, women’s needs to function as infant mothers must be considered in the postpartum treatment of BD.

### Strengths and limitations

This study’s foremost strength is its mixed methods design, which allows quantitative data on the occurrence of mood symptoms to be combined with rich qualitative descriptions of the subjective experiences of infant mothers with BD. However, there are limitations to note. The women’s retrospective reports on mood deviations, apart from the specific IDS and YMRS assessments at 3 and 12 months, may have been influenced by recollection bias. On the other hand, the occurrences of mood deviations were thoroughly explored in the interviews with in-depth descriptions and were often anchored to important events during the first year, such as baptism, holidays, the return to work and being on sick leave.

There are indications of skewed resourcefulness in our sample. The women all had a cohabitating partner and were satisfied with their life situation, and several had access to familial support. These circumstances call for caution in generalising the results, since the findings might have been different with more diversity in the sample, such as single mothers. However, the fact that our thematic findings on challenges and ways of promoting wellbeing replicate and overlap with findings across studies from other parts of the world with diverse samples supports the validity of the findings.

Departing from many qualitative reports, we used quantification of the qualitative data to convey accurate occurrences of the viewpoints and make the basis for our interpretative claims more transparent. Nevertheless, the viewpoints were spontaneously expressed in the interviews, and there is the possibility that more women had similar views without expressing them.

## Electronic supplementary material

Below is the link to the electronic supplementary material.

Supplementary Material 1

Supplementary Material 2

## Data Availability

Raw data will not be shared or made publicly available, since participants may be identifiable. Informed consent for this was not sought from the participants prior to data collection. Requests for permission to access data may be sent to the corresponding author.

## Footnotes

1 Henceforth, all time specifications in the text, i.e., first year, three months and 12 months, refer to the postpartum period.

2 Mental health service for prevention of mother–infant relationship difficulties.

3 IDS score 34 and YMRS score 16 at 12 months.

4 Standardised assessments conducted by external independent coders.

## Abbreviations

BD: Bipolar disorder
Pos. Gr: Positive group in terms of main perception of the first year
Mix. Gr: Mixed group in terms of main perception of the first year
