# Supplementary material for: “Bad timing for illness relapse!” Mood symptoms, challenges and strategies for wellbeing in the first year postpartum among infant mothers with bipolar disorder: a mixed-methods study
Source: Int J Bipolar Disord. 2025 Feb 24;13:9. doi: 10.1186/s40345-025-00374-x (PMC11850689; doi:10.1186/s40345-025-00374-x)
Supplement: Supplementary file 1 — Supplementary Material 1 [file 40345_2025_374_MOESM1_ESM.docx]

**Supplementary file 1.** Information on type and dosage of medication for 26 women with BD, organised into four subgroups based on symptom severity at 3 and 12 months postpartum.

**Supplementary Table 1.1** Participants’ (N=26) medication and dosage at 1, 3 and 12 months postpartum

| **Subgroup** | **Parti-**  **cipant** | **Medication**  **1 month**  **postpartum** | **Medication**  **3 months**  **postpartum** | **Medication**  **12 months**  **postpartum** |
| --- | --- | --- | --- | --- |
| **1.**  **Euthymic**  **or**  **mild symptoms** | 1 BD I | Olanzapine 15 mg | Olanzapine 12,5 mg | Olanzapine 12,5 mg |
|  | 2 BD I | Lithium 42 + 84 mg  Quetiapine 25 mg  Escitalopram 5 mg  Zopiclone 5 mg | Lithium 83 mg x 2  Escitalopram 5 mg | Lithium 83 mg x 2  Escitalopram 5 mg |
|  | 3 BD II | Lamotrigine 200 mg | Lamotrigine 200 mg | Lamotrigine 200 mg |
|  | 4 BD II | Lamotrigine 300 mg | Lamotrigine 300 mg | Lamotrigine 300 mg |
|  | 5 BD II | None | None | None |
|  | 6 BD II | Lamotrigine 200 mg | Lamotrigine 200 mg | Lamotrigine 200 mg |
|  | 7 BD I | Lamotrigine 400 mg | Lamotrigine 400 mg | Lamotrigine 400 mg |
|  | 8 BD II | None | None | None |
|  | 9 BD II | Lamotrigine 800-> 300 mg | Lamotrigine 300 mg | Lamotrigine 400 mg  Escitalopram 10 mg |
|  | 10 BD II | Lamotrigine 200 mg  Venlafaxine 37,5 mg | Lamotrigine 200 mg  Venlafaxine 37,5 mg | Lamotrigine 200 mg  Venlafaxine 37,5 mg |
|  | 11 BD II | None | None | None |
| **2.**  **At least one time point with moderate**  **affective**  **symptoms** | 12 BD II | None | None | None |
|  | 13 BD II | Lamotrigine 500 mg  Bupropion 300 mg | Lamotrigine 500 mg  Bupropion 300 mg | Lamotrigine 500 mg  Bupropion 300 mg |
|  | 14 BD II | Lamotrigine 300 mg  Sertraline 100 mg | Lamotrigine 300 mg  Sertraline 100 mg | Lamotrigine 300 mg  Sertraline 150 mg |
|  | 15 BD II | None | None | None |
|  | 16 BD II | Bupropion 150 mg  Risperidone 0,5 mg | Bupropion 150 mg  Risperidone 0,5 mg  Pregabalin 125 mg | Bupropion 300 mg  Risperidone 0,25 mg  Pregabalin PRN |
|  | 17 BD II | Lamotrigine 200 mg  Quetiapine 25 mg  Zopiclone 5 mg | Lamotrigine 200 mg Quetiapine 37,5 mg  Zopiclone 5 mg | Lamotrigine 600 mg  Escitalopram 10 mg  Zopiclone 5 mg |
| **3.**  **At least one time point with**  **severe**  **affective**  **symptoms** | 18 BD I | Lithium 83 mg Lamotrigine 200 mg  Escitalopram 5 mg | Lithium 83 mg Lamotrigine 200 mg  Escitalopram 5 mg | Lithium 83 mg Lamotrigine 200 mg  Escitalopram 10 mg |
|  | 19 BD II | None | Flupentixol 2 mg | Flupentixol 2 mg Lamotrigine 150 mg  Bupropion 150 mg |
|  | 20 BD II | None | None | None |
|  | 21 BD II | None | None | None |
|  | 22 BD II | Lithium 42 + 84 mg  Quetiapine 100 mg  Bupropion 150 mg  Sertraline 100 mg | Lithium 84 mg x 2  Quetiapine 100 mg  Sertraline 25 mg  Oxazepam 5 mg PRN | Lithium 84 mg x 2 Quetiapine 150 mg  Sertraline 100 mg  Oxazepam 5 mg PRN |
|  | 23 BD II | Lamotrigine 300 mg | Lamotrigine 300 mg | Lamotrigine 400 mg |
| **4.**  **Psychosis** | 24 BD I | Lithium 42 + 84 mg  Escitalopram 10 mg | Lithium 42 + 84 mg  Escitalopram 10 mg | None. Ceased medication at 10 months |
|  | 25 BD I | Lithium 84 mg x 2 | Lithium 84 mg x 2 | Lithium 84 mg x 2 |
|  | 26 BD I | Lithium 84 + 126 mg  Olanzapine 15 + 10 mg | Lithium 42 + 126 mg  Olanzapine 10 mg | Lithium 126 mg x 2  Olanzapine 10 mg x 2 |
