# Supplementary material for: “Bad timing for illness relapse!” Mood symptoms, challenges and strategies for wellbeing in the first year postpartum among infant mothers with bipolar disorder: a mixed-methods study
Source: Int J Bipolar Disord. 2025 Feb 24;13:9. doi: 10.1186/s40345-025-00374-x (PMC11850689; doi:10.1186/s40345-025-00374-x)
Supplement: Supplementary file 2 — Supplementary Material 2 [file 40345_2025_374_MOESM2_ESM.docx]

**Supplementary File 2.** Information on scores at 3 and 12 months on extracted IDS items and on YMRS.

Supplementary table 2.1 shows the distribution of scores on nine extracted IDS items in 25^[[1]](#footnote-1)^ women with BD, organised into four subgroups based on symptom severity (i.e. when all 30 IDS items are included) at 3 and 12 months postpartum. The extracted items are chosen based on them being key depressive symptoms and/or relevant for the thematic findings in the current study.

**Supplementary Table 2.1.** Participants’ (n=25) scores on nine IDS items at 3 and 12 months.

| **Item, number**  **and scoring** | **Subgroup 1**  **Euthymic**  **or mild symptoms**  **N=11**  **3 months 12 months**  **n % n %** | | **Subgroup 2**  **At least one timepoint with moderate**  **affective symptoms**  **N=6**  **3 months 12 months**  **n % n %** | | **Subgroup 3**  **At least one timepoint with severe**  **affective symptoms**  **N=6**  **3 months 12 months**  **n % n %** | | **Subgroup 4**  **Psychosis**  **N=2**  **3 months 12 months**  **n % n %** | |
| --- | --- | --- | --- | --- | --- | --- | --- | --- |
| **Feeling sad (5)** | **6 54** | **4 36** | **5 83** | **6 100** | **4 67** | **6 100** | **2 100** | **2 100** |
| 1- Less than half of the time | 6 54 | 4 36 | 4 67 | 4 67 | 2 33 | - | - | 2 100 |
| 2 - More than half of the time | - | - | 1 17 | 2 33 | 2 33 | 6 100 | 2 100 | - |
| **Feeling irritable (6)** | **8 73^2^** | **8 73** | **6 100** | **5 83** | **4 67** | **6 100** | **2 100** | **1 50** |
| 1 - Less than half of the time | 6 54 | 7 64 | 5 83 | 3 50 | 2 33 | 3 50 | 1 50 | - |
| 2 - More than half of the time | 1 9 | 1 9 | 1 17 | 2 33 | 1 17 | 3 50 | 1 50 | 1 50 |
| 3 - Extremely irritable nearly all the time | 1 9 | - | - | - | 1 17 | - | - | - |
| **Feeling anxious or tense (7)** | **5 45** | **4 36** | **4 67** | **6 100** | **4 67** | **4 67** | **1 50** | **2 100** |
| 1 - Less than half of the time | 5 45 | 4 36 | 2 33 | 2 33 | 3 50 | 3 50 | 1 50 | 1 50 |
| 2 - More than half of the time | - | - | 1 17 | 2 33 | 1 17 | 1 17 |  | 1 50 |
| 3 - Extremely anxious (tense) nearly all  of the time. | - | - | 1 17 | 2 33 | - | - | - | - |
| **Concentration/Decision Making (15**) | **5 45** | **2 18** | **4 67** | **6 100** | **3 50** | **6 100** | **1 50** | **1 50** |
| 1 - Occasionally feels indecisive or finds  that attention wanders | 4 36 | 1 9 | 3 50 | 2 33 | 1 17 | 4 67 | - | - |
| 2 - Most of the time, struggles to focus  attention or to make decisions | - | 1 9 | 1 17 | 4 67 | 2 33 | 1 17 | 1 50 | 1 50 |
| 3 - Cannot concentrate well enough to read  or cannot make even minor decisions. | 1 9 | - | - | - | - | 1 17 | - | - |
| **View of self (16)** | **4 36** | **1 9** | **4 67** | **5 83** | **3 50** | **5 83** | **2 100** | **2 100** |
| 1 - More self-blaming than usual. | 4 36 | 1 9 | 3 50 | 3 50 | 2 33 | 2 33 | - | 1 50 |
| 2 - Largely believes that one causes  problems for others. | - | - | 1 17 | 1 17 | - | 1 17 | 1 50 | - |
| 3 - Almost constantly thinking about major  and minor defects in oneself | - | - | - | 1 17 | 1 17 | 2 33 | 1 50 | 1 50 |
| **General Interest (19)** | **2 18** | **3 27** | **3 50** | **4 67** | **3 50** | **5 83** | **2 100** | **2 100** |
| 1 - Less interest in people or activities | 1 9 | 3 27 | 3 50 | 4 67 | 3 50 | 3 50 | 2 100 | 1 50 |
| 2 - Interest in only one or two of formerly  pursued activities | 1 9 | - | - | - | - | 2 33 | - | 1 50 |
| **Energy Level (20)** | **2 18** | **2 18** | **3 50** | **6 100** | **4 67** | **6 100** | **2 100** | **2 100** |
| 1 - Gets tired more easily than usual | 2 18 | 2 18 | 3 50 | 3 50 | 1 17 | 1 17 | 1 50 | 1 50 |
| 2 - Has to make a big effort to start or finish usual  daily activities (for example, shopping,  homework, cooking or going to work). | - | - | - | 3 50 | 3 50 | 5 83 | 1 50 | 1 50 |
| **Feeling slowed down (23)** | **1 9** | **1 9** | **1 17** | **4 67** | **2 33** | **4 67** | **2 100** | **0** |
| 1 - Finds that thinking is slowed down or  voice sounds dull or flat | 1 9 | 1 9 | 1 17 | 4 67 | 2 33 | 4 67 | 2 100 | - |
| **Interpersonal Sensitivity (29)** | **5 45** | **4 36** | **2 33** | **4 67** | **4 67** | **5 83** | **2 100** | **2 100** |
| 1 - Has occasionally felt rejected, slighted,  criticized or hurt by others | 3 27 | 2 18 | - | 2 33 | 3 50 | 1 17 | - | - |
| 2 - Has often felt rejected, slighted, criticized or  hurt by others, but these feelings have had only  slight effects on relationships or work | 2 18 | 2 18 | 2 33 | 2 33 | - | 4 67 | 2 100 | 2 100 |
| 3 - Has often felt rejected, slighted, criticized or  hurt by others and these feelings have impaired  relationships and work | - | - | - | - | 1 17 | - | - | - |

Supplementary table 2.2 shows the distribution of scores on YMRS in four^3^ women with BD.

**Supplementary Table 2.2.** Scores on YMRS for participants with hypomanic symptoms (N=4), at 3 months (n=3) and 12 months (n=1)

| **Item and score** | **Number of participants receiving the score** |
| --- | --- |
| **1: Elevated Mood** |  |
| 1: Mildly or possibly increased questioning | 1/4 |
| 2: Definite subjective elevation | 3/4 |
| **2: Increased Motor Activity-Energy** |  |
| 1: Subjectively increased | 1/4 |
| 2: Animated; gestures increased | 3/4 (one was rated 1,5) |
| **3: Sexual Interest** |  |
| 1: Mildly or possibly increased | 1/4 |
| **4: Sleep** |  |
| 1: Sleeping less than the normal amount by up  to one hour | 1/4 |
| 3: Reports decreased need for sleep | 2/4 |
| **5: Irritability** |  |
| 2: Subjectively increased | 3/4 |
| **6: Speech (Rate and Amount)** |  |
| 2: Feels talkative | 1/4 |
| 4: Increased rate or amount at times, verbose  at times | 3/4 |
| **7: Language-Thought Disorder** |  |
| 1: Circumstantial; mild distractibility; quick  thoughts | 1/4 |
| 2: Distractible, loses goal of thought; changes  topics frequently; racing thoughts | 2/4 |
| **8: Content** |  |
| 2**:** Questionable plans, new interests | 2/4 |
| **9: Disruptive-Aggressive Behavior** | 0/4 |
| **10: Appearance** |  |
| 1: Minimally unkempt | 1/4 |
| 2: Poorly groomed; moderately disheveled;  overdressed | 1/4 |
| **11: Insight** |  |
| 1**:** Possibly ill | 2/4 |

^3^ Missing data on one participant on YMRS because of psychosis and therefore not included in Supplementary table 2.2.

1. Missing data on one participant on IDS because of psychosis and therefore not included in Supplementary table 2.1.

   ^2^ Deviations between single and total percentage numbers are due to rounding of decimals that are not shown. [↑](#footnote-ref-1)
